# Nitric oxide in occurrence, progress and therapy of lung Cancer: a systemic review and meta-analysis

**DOI:** 10.1186/s12885-021-08430-2

**Published:** 2021-06-08

**Authors:** Hongbin Zhou, Jiuke Li, Zhewen Chen, Ying Chen, Sa Ye

**Affiliations:** 1 Department of Respiratory Medicine, Department of Nutrition, Zhejiang Provincial People’s Hospital, Affiliated People’s Hospital, Hangzhou Medical College, 158 Shangtang Road, Hangzhou, 310014 China; 2 Department of Ophthalmology, Hangzhou Aier Eye Hospital, Zhejiang, Hangzhou China; 3 Department of Nutrition, Zhejiang Provincial People’s Hospital, Affiliated People’s Hospital, Hangzhou Medical College, Zhejiang, Hangzhou China

**Keywords:** Nitric oxide (NO), Fraction of exhaled nitric oxide (FeNO), Lung cancer

## Abstract

**Background:**

Nitric oxide (NO) plays an important role in lung cancer. However, the results of previous studies about NO in the occurrence, progress and therapy were not consistent. Therefore, we conducted a meta-analysis to evaluate the relationship between NO and lung cancer.

**Method:**

We carried out comprehensive search in the databases, and collected related studies. The data of fraction of exhaled nitric oxide (FeNO) or blood NO in different populations (lung cancer patients and control subjects) and different time points (before therapy and after therapy) were extracted by two investigators. A random effect model was applied to analyze the differences of FeNO and blood NO in different populations and different time points. To further compare NO level of each subgroup with different pathological types and different stages, a network meta-analysis (NMA) was performed.

**Results:**

Fifty studies including 2551 cases and 1691 controls were adopted in this meta-analysis. The FeNO (SMD 3.01, 95% CI 1.89–4.13, *p* < 0.00001) and blood NO (SMD 1.34, 95% CI 0.84–1.85, *p* < 0.00001) level in lung cancer patients was much higher than that in control subjects. NMA model indicated blood NO level in each cancer type except SCLC was higher than that in control patients. There was no significant difference of blood NO level among four kinds of lung cancer patients. Blood NO level in LCC patients (SUCRA = 83.5%) was the highest. Blood NO level in advanced stage but not early stage was higher than that in control subjects. Patients in advanced stage (SUCRA = 95.5%) had the highest blood NO level. No significant difference of FeNO (SMD -0.04, 95% CI -0.46-0.38, *p* > 0.05) and blood NO level (SMD -0.36, 95% CI -1.08-0.36, *p* > 0.05) was observed between pretreatment and posttreatment in all patients. However, FeNO level elevated (SMD 0.28, 95% CI 0.04–0.51, *p* = 0.02) and blood NO level decreased in NSCLC patients (SMD -0.95, 95% CI -1.89-0.00, *p* = 0.05) after therapy.

**Conclusion:**

FeNO and blood NO level would contribute to diagnosis of lung cancer and evaluation of therapy effect, especially for NSCLC patients.

**Supplementary Information:**

The online version contains supplementary material available at 10.1186/s12885-021-08430-2.

## Introduction

Lung cancer, a kind of tumor with the highest incidence, is also the leading cause of cancer death in the world [1]. It was estimated that about 1.76 million patients died from lung cancer in 2018 globally [2]. The pathogenesis of lung cancer is considered as a multi-stage process, which is affected by genetic background and environmental factors [3]. So far, the underlying mechanisms have not been fully understood. However, it has been widely accepted that oxidative/nitrative damage is the crucial component element leading to carcinogenesis.

Nitric oxide (NO), a small molecule derived from L-arginine, has been demonstrated to participate in inflammation, tumor immunity and tumor apoptosis, as well as other pathophysiological process, which are associated with the pathogenesis, progression of lung cancer [4]. NO is produced by airway epithelial cells, vascular endothelial cells, resident macrophages and recruiting inflammatory cells in respiratory system. A part of NO in the airway will be expired through breath movement, and the fraction of exhaled nitric oxide (FeNO) can be measured by a specific equipment. The value of FeNO is considered as an indicator of airway inflammation level and applied in other chronic respiratory diseases, such as asthma and COPD [5]. In spite of gaseous form, NO also exists in the forms of nitrate or nitrite in circulatory system. The concentration of nitrate or nitrite in peripheral blood can be detected to evaluate systemic NO level.

During the past several decades, many researchers carried out a series of studies try to clarify the relationship between NO and lung cancer. These studies were mainly about comparisons of the levels of FeNO and blood NO between lung cancer patients and healthy controls [6–45], among lung cancer patients with different stages [7–9, 14, 17–21, 23, 27, 29, 31, 34, 36–39, 42] or types [6, 8, 9, 18–21, 24, 26, 27, 29, 31, 37–39, 42], and variations of these indexes from pretreatment to posttreatment [7, 10, 14, 19, 21, 29, 32, 34, 36, 38, 39, 43, 46–55]. However, the results were not consistent among different studies, which might be due to ethnicity, age, pathological type, cancer stage, treatment regimen and follow up period. So, we conducted this meta-analysis to investigate the role of NO in occurrence, progress and therapy of lung cancer.

## Methods

### Search strategy

We adopted a comprehensive search strategy in databases, including PubMed, Embase and CNKI, to identify the studies about NO and lung cancer. The terms we used were as follows:” lung cancer”,” nitric oxide”, “NO”, “fraction of exhaled nitric oxide”, “FeNO”. Additional studies were identified by a manual search from references of original studies or review articles on this topic.

### Study selection

The criteria for the selection of studies were as follows: (1) Case-control studies with control group and at least one case group consisting of lung cancer patients; (2) Other observational studies about comparison between pretreatment and posttreatment, regardless of concrete therapeutic regimen; (3) Case-control studies should provide FeNO or blood NO data from both control group and case group; (4) Other observational studies should provide FeNO or blood NO data collected before and after treatment. The studies including other kinds of cancers, in which the data of lung cancer could not be obtained alone, were excluded from our analysis.

### Data extraction

The data were independently extracted from all eligible publications by two authors according to the inclusion criteria listed above. Any disagreements were resolved by discussions with a third person. The information extracted from all the studies included author, publication year and observed indexes (FeNO, blood NO). For case-control studies, the traits of enrolled patients and control subjects (quantity, nationality, sex, age, smoking status), and cancer characteristics (histological type and stage) were also involved. For other observational studies, the basic and therapy information (quantity, nationality, sex, age, histological type, stage, regimen and follow up period) of enrolled patients were recorded.

### Quality assessment

All the included studies were assessed with the help of Newcastle-Ottawa Scale (NOS), which was mainly about three aspects composed of selection, comparability and exposure [56]. Each study was assigned a score from 0 to 9 points, and higher points indicated higher quality.

### Statistical analysis

Standard mean difference (SMD) and 95% confidence intervals (CIs) were used to assess the differences of FeNO and blood NO concentrations between control subjects and lung cancer patients, or between pretreatment and posttreatment.

I^2^ statistic was used to quantify the degree of heterogeneity, with I^2^ < 25, 25–75% and > 75% representing low, moderate and high degrees of inconsistency, respectively [57, 58]. In the analysis of pooled data, we used two different models according to the traits of the included studies: If no heterogeneity was found, a fixed effect model was adopted to determine the effects or a random effect model was applied. If heterogeneity existed across studies, a subgroup analysis was performed to seek out the source of heterogeneity. The studies were subdivided by nationality (eastern countries vs. western countries or China vs. Non-China), Age (matched vs. unmatched vs. unknown), sex proportion (matched vs. unmatched vs. unknown), histological types (NSCLC vs. NSCLC + SCLC vs. unknown), stages (early + advanced vs. early vs. advanced vs. unknown), treatment (surgery vs. chemotherapy vs. radiotherapy vs. other therapy), and follow up period (long term vs. short term vs. unknown).

To further compare NO level of each subgroup with different pathological types and different stages, a network meta-analysis (NMA) was conducted. NMA was performed through a Bayesian random-effect model with Markov Chain Monte Carlo simulations which was executed by the ‘gemtc’ package in R. Using four parallel chains, 50,000 samples after 20,000-sample burn-in were obtained in each chain. Convergence of the model was evaluated with the use of Brooks-Gelman-Rubin diagnostic method in the ‘coda’ package in R. Consistency of the NMA, defined as a statistical discrepancy between direct and indirect comparison results, was assessed with the help of a node-splitting approach in the ‘gemtc’ package in R.

The model ranked each pathological type or stage by their relative effect (probabilities of being higher). Cumulative probability of being the pathological type or stage with the highest NO level was calculated. With that, the surface under the cumulative ranking curve (SUCRA) of each pathological type or stage is obtained [59]. Specifically, SUCRA is a numeric presentation of the overall ranking and presents a single number, ranging from 0 to 100%, associated with each subgroup, where 0% represents the lowest NO level and 100% represents the highest NO level.

We made use of a Begg’s funnel plot to examine the underlying publication bias and Egger’s weighted regression method to calculate a *P* value for bias [60, 61]. If no publication bias exists, the funnel plot appears symmetrical.

All analyses were conducted with the use of Review Manager, V.5.2 (Revman, The Cochrane Collaboration, London, UK), STATA software, V.14.0 (StataCorp LP, College Station, TX, USA) and R software, V.4.0.3.

## Results

### Characteristics of the included studies

**Fig. 1:**
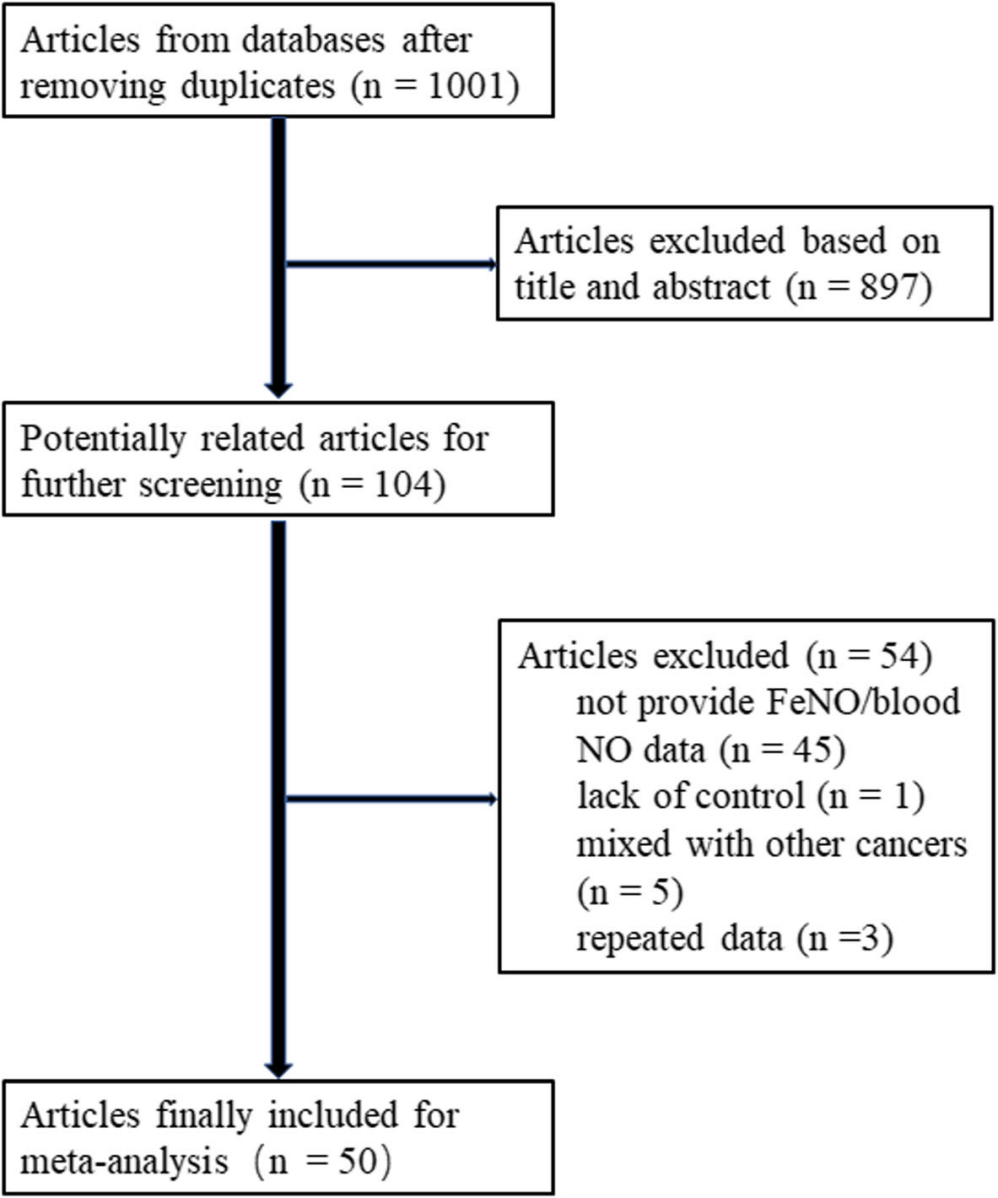
The flowchart of the study selection process for the meta-analysis

We identified 1001 related articles, of which 104 studies were potentially suitable. Forty-five studies were eliminated because they provided neither FeNO nor blood NO data. One study was excluded due to lack of control subjects. Five studies were ruled out because these studies contained different kinds of cancer patients, and the data of lung cancer could not be obtained. In addition, 3 repeated studies were also discarded. Finally, 50 studies including 2551 cases and 1691 controls met the including criteria (Fig. 1). There were 40 case-control studies, in which 6 studies were about FeNO, while 34 studies showed blood NO data. Seven studies provided FeNO data, while 15 studies provided blood NO data collected before and after treatment, respectively. The study characteristics were listed in Table 1, 2, 3 and 4. Lung cancer patients were diagnosed by operation or biopsy. Frequency-matched controls to the cases by sex, age and smoking status were applied in some studies. The treatment regimen mentioned in these studies included operation, chemotherapy, radiotherapy, immunotherapy and traditional Chinese medicine. The scores of included studies ranged from 5 to 9 by NOS.

**Table 1:** Characteristics of case-control studies about FeNO

Year	Author	Country	Age matched	Sex matched	Smoking matched	Histology	Stage	FeNO (ppb)				NOS
								Control		Case		
								Mean ± SD	n	Mean ± SD	n	
1998	Liu	China	NA	Yes	Yes	SCC, ADC, LCC, SCLC	advanced	6.0 ± 0.5	20	16.9 ± 0.9	28	7
2001	Koizumi	Japan	NA	NA	NA	SCC, ADC, LCC, SCLC	early + advanced	44 ± 13	11	77 ± 47	29	5
2005	Masri	USA	NA	NA	NA	mixed (mainly SCC, ADC)	NA	7.4 ± 0.54	35	18.4 ± 3.16	11	5
2016	Xu	China	Yes	Yes	Yes	SCC, ADC, SCLC	early + advanced	16.12 ± 5.85	51	25.60 ± 16.93	61	9
2018	Feng	China	Yes	Yes	Yes	SCC, ADC, SCLC	NA	16.8 ± 4.1	50	33.8 ± 15.6	50	8
2018	Liu	China	Yes	Yes	NA	SCC, ADC, SCLC	NA	16.83 ± 4.17	172	33.85 ± 15.63	164	8

*Abbreviations*: *NA* Not available, *NOS* Newcastle-Ottawa Scale, *SCC* Squamous cell carcinoma, *ADC* Adenocarcinoma, *LCC* Large cell carcinoma, *SCLC* Small cell lung cancer

**Table 2:** Characteristics of case-control studies about blood NO

Year	Author	Country	Age matched	Sex matched	Smoking matched	Histology	Stage	Blood NO (μmol/L)				NOS
								Control		Case		
								Mean ± SD	n	Mean ± SD	n	
1996	Ma	China	Yes	Yes	NA	SCC, ADC, LCC, SCLC	NA	34.8 ± 18.2	34	48.5 ± 15.8	28	5
1997	Fan	China	Yes	Yes	NA	mixed (mainly SCC, ADC)	early + advanced	29.32 ± 16.78	25	58.75 ± 19.63	25	5
1998	Li	China	NA	Yes	NA	SCC, ADC, SCLC	early + advanced	42.10 ± 17.22	18	91.78 ± 47.25	25	6
1998	Yu	China	Yes	Yes	NA	SCC, ADC	early + advanced	5.80 ± 1.00	30	7.11 ± 1.35	46	6
1999	Wu	China	Yes	Yes	NA	SCC, ADC, SCLC	early + advanced	29.6 ± 11.4	30	37.8 ± 14.1	30	7
1999	Yu	China	Yes	Yes	NA	SCC, ADC	early	5.82 ± 1.31	25	9.91 ± 1.03	18	6
2001	Bao	China	No	No	NA	SCC, ADC, SCLC	early + advanced	67.64 ± 32.05	40	53.22 ± 26.08	57	6
2001	Song	China	NA	Yes	NA	SCC, ADC, SCLC	early + advanced	3.45 ± 0.94	28	4.93 ± 2.58	30	6
2001	Zhu	China	No	No	NA	SCC, ADC, SCLC	early + advanced	84.69 ± 12.10	17	119.37 ± 13.91	40	5
2001	zheng	China	Yes	Yes	NA	SCC, ADC, LCC, SCLC	early + advanced	73.42 ± 13.56	30	49.42 ± 18.78	40	6
2002	Wu	China	NA	NA	NA	SCC, ADC, SCLC	early + advanced	85.54 ± 39.63	20	48.69 ± 39.32	40	5
2002	Wang	China	Yes	Yes	NA	SCC, ADC	early + advanced	2.016 ± 0.524	36	5.902 ± 1.157	48	6
2002	Shi	China	NA	Yes	NA	NA	NA	2.012 ± 0.869	29	6.952 ± 2.571	17	6
2002	Zhao	China	No	No	NA	SCC, ADC	early + advanced	5.48 ± 1.06	60	7.38 ± 2.15	41	5
2002	Liu	China	No	Yes	NA	NSCLC, SCLC	early + advanced	49.3 ± 17.9	20	89.7 ± 25.0	70	6
2002	Li	China	No	Yes	NA	SCC, ADC	NA	5.24 ± 1.18	40	7.48 ± 1.65	30	6
2003	Liu	China	Yes	No	NA	SCC, ADC	NA	5.26 ± 1.14	50	8.65 ± 2.06	36	6
2003	Yu	China	Yes	Yes	Yes	SCC, ADC	early + advanced	78.38 ± 15.78	30	105.71 ± 25.68	30	7
2003	Zhao	China	NA	Yes	NA	NA	early	24.2 ± 10.4	35	60.4 ± 27.2	40	6
2004	Tang	China	NA	Yes	NA	SCC, ADC, LCC	NA	76.81 ± 16.61	40	122.3 ± 26.21	51	7
2005	Fu	China	NA	Yes	NA	SCC, ADC, LCC	early + advanced	44.2 ± 15.0	20	237.1 ± 21.0	82	7
2005	Yin	China	Yes	Yes	NA	SCC, ADC, SCLC	early + advanced	4.77 ± 1.81	10	35.59 ± 25.47	41	6
2005	Chen	China	NA	NA	NA	NA	early	68.5 ± 14.7	35	30.4 ± 12.9	31	5
2005	Kaynar	Turkey	Yes	Yes	NA	NSCLC, SCLC	early + advanced	43.4 ± 9.3	16	53.9 ± 8.8	32	6
2006	Colakogullari	Turkey	NA	NA	NA	NSCLC, SCLC	advanced	63.7 ± 32.2	15	93 ± 48	31	6
2006	Li	China	Yes	Yes	NA	SCC, ADC	early + advanced	54.6 ± 16.4	60	77.1 ± 22.3	62	6
2008	Yan	China	NA	No	NA	NA	NA	23.67 ± 6.18	30	21.97 ± 9.68	40	5
2008	Esme	Turkey	Yes	No	Yes	SCC, ADC, LCC	early + advanced	145.93 ± 27.03	20	211.32 ± 70.11	49	7
2009	Long	China	NA	Yes	NA	SCC, ADC, LCC	NA	77.81 ± 17.61	80	123.30 ± 25.21	100	6
2010	Srivastava	India	Yes	Yes	NA	mainly SCC, ADC, LCC	advanced	11.88 ± 3.48	144	25.76 ± 4.54	203	8
2012	He	China	No	No	NA	SCC, ADC	early + advanced	79.30 ± 10.88	50	125.89 ± 10.78	40	6
2014	Zhang	China	Yes	Yes	Yes	NSCLC	NA	16.79 ± 5.37	100	19.68 ± 4.11	110	9
2015	Xu	China	NA	NA	NA	SCC, ADC, LCC, SCLC	NA	68.4 ± 12.6	35	30.1 ± 11.7	36	5
2020	Bayraktutan	Turkey	Yes	Yes	No	NSCLC, SCLC	NA	24.03 ± 12.61	100	30.95 ± 18.20	100	8

*Abbreviations*: *NA* Not available, *NOS* Newcastle-Ottawa Scale, *SCC* Squamous cell carcinoma, *ADC* Adenocarcinoma, *LCC* Large cell carcinoma, *NSCLC* Non-small cell lung cancer, *SCLC* Small cell lung cancer

**Table 3:** Characteristics of studies about FeNO variation in lung cancer patients between pretreatment and posttreatment

Year	Author	Country	Age (Mean ± SD)	Sex (M/F)	Smoking status (Mean ± SD)	Histology	Stage	Treatment	Follow up period	FeNO (ppb)			
										Before therapy		After therapy	
										Mean ± SD	n	Mean ± SD	n
2001	Koizumi	Japan	61^a^ (38–84)	24/5	NA	SCC, ADC, LCC, SCLC	early + advanced	radio-therapy	NA	77.0 ± 47.0	29	49.0 ± 33.0	29
2006	Wewel	Germany	64 (12)^b^	27/12	40 (29)^b^	NSCLC, SCLC	early + advanced	chemo-therapy	21–29 days	22.1 ± 10.1	34	24.5 ± 10.8	34
2012	Enache	France	61 ± 11	48/17	NA	SCC, ADC, LCC, SCLC	early + advanced	radio-therapy	7.5 months	14.3 ± 7.2	65	18.2 ± 12.5	65
2013	Kallianos	Greece	65 ± 17	26/16	51 ± 7	SCC, ADC, LCC, SCLC	NA	chemo-therapy	NA	9.8 ± 2.1	42	7.7 ± 1.6	42
2014	Moré	USA	68^a^ (54–89)	14/6	NA	NSCLC	NA	radio-therapy	6 months	26.3 ± 19.6	17	31.8 ± 22.9	17
2019	Szejniuk	Denmark	66^a^ (40–78)	24/18	NA	NSCLC	early + advanced	radio-therapy	12 months	13.5 ± 7.6	42	16.8 ± 9.8	42
2019	Suzuki	Japan	69^a^ (65–74)	74/21	NA	NSCLC	advanced	immune-therapy	NA	21.5 ± 14.7	85	25.2 ± 16.8	85

*Abbreviations*: *NA* Not available, *SCC* Squamous cell carcinoma, *ADC* Adenocarcinoma, *LCC* Large cell carcinoma, *NSCLC* Non-small cell lung cancer, *SCLC* Small cell lung cancer

^a^Data presented as median (range)

^b^Data presented as median (interquartile range)

**Table 4:** Characteristics of studies about blood NO variation in lung cancer patients between pretreatment and posttreatment

Year	Author	Country	Age (Mean ± SD)	Sex (M/F)	Smoking status	Histology	Stage	Treatment	Follow up period	Blood NO (μmol/L)			
										Before therapy		After therapy	
										Mean ± SD	n	Mean ± SD	n
1997	Zhang	China	58.4^a^	13/7	NA	SCC, ADC, LCC, SCLC	advanced	chemo-therapy	1 week	51.2 ± 16.9	10	79.8 ± 21.3	10
1998	Yu	China	64.2^a^	28/18	NA	SCC, ADC	early + advanced	surgery	NA	7.51 ± 1.40	26	6.68 ± 1.19	26
								chemo-therapy	NA	6.58 ± 1.30	15	5.43 ± 1.43	15
1999	Yu	China	49 士 3.8	14/4	NA	SCC, ADC	early + advanced	chemo-therapy /surgery	NA	9.91 ± 1.03	18	8.48 ± 1.06	18
2002	Li	China	64.3^b^ (37–75)	53/31	NA	SCC, ADC	early + advanced	chemo-therapy	NA	64.6 ± 29.5	84	88.9 ± 39.4	84
2002	Wang	China	65.4^a^	35/13	NA	SCC, ADC, LCC	early + advanced	surgery	NA	7.124 ± 1.631	26	6.094 ± 0.917	26
2003	Liu	China	61.4 ± 11.3	30/6	NA	SCC, ADC	NA	surgery	10 days	8.72 ± 1.96	20	6.83 ± 1.75	20
2003	Zhao	China	51.8^a^	28/12	NA	NA	early	surgery	1 month	60.4 ± 27.2	40	37.8 ± 15.1	40
2005	Yin	China	57.04 ± 11.87	33/8	NA	SCC, ADC, SCLC	early + advanced	chemo-therapy	2 weeks	38.03 ± 36.59	40	29.33 ± 20.97	40
2005	Chen	China	NA	NA	NA	NA	early	surgery	3 months	30.4 ± 12.9	31	62.2 ± 15.1	30
2006	Li	China	57.8^a^	47/15	NA	SCC, ADC	early + advanced	surgery	21 days	86.7 ± 24.1	19	84.7 ± 18.4	19
								chemo-therapy	21 days	89.4 ± 22.1	27	55.7 ± 16.7	27
2006	Colakogullari	Turkey	58.5 ± 10.6	25/6	NA	NSCLC, SCLC	advanced	chemo-therapy	2 days	93.0 ± 48.0	31	106.0 ± 49.0	16
2010	Srivastava	India	55^b^ (30–88)	155 / 48	NA	mainly SCC, ADC, LCC	advanced	chemo-therapy	88 weeks	25.76 ± 4.54	203	32.81 ± 4.27	155
2015	Xu	China	36–80^c^	24/12	NA	SCC, ADC, LCC, SCLC	NA	chemo-therapy	NA	30.1 ± 11.7	36	62.0 ± 14.7	36
2017	Muto	Japan	65.3 ± 6.3	6/9	NA	ADC	advanced	chemo-therapy	> 30 months	62.7 ± 40.0	15	37.6 ± 27.5	15
2019	Feng	China	52.7 ± 4.6	28/15	NA	SCC, ADC	advanced	chemo-therapy	3 months	125.08 ± 14.89	43	89.77 ± 11.35	43
			53.4 ± 3.6	26/18	NA	SCC, ADC	advanced	chemo-therapy + TCM	3 months	126.56 ± 14.57	44	78.12 ± 8.12	44

*Abbreviations*: *NA* Not available, *SCC* Squamous cell carcinoma, *ADC* Adenocarcinoma, *LCC* Large cell carcinoma, *NSCLC* Non-small cell lung cancer, *SCLC* Small cell lung cancer

^a^Data presented as mean value

^b^Data presented as median (range)

^c^Data presented as range

### FeNO level between lung cancer patients and control subjects

**Fig. 2:**
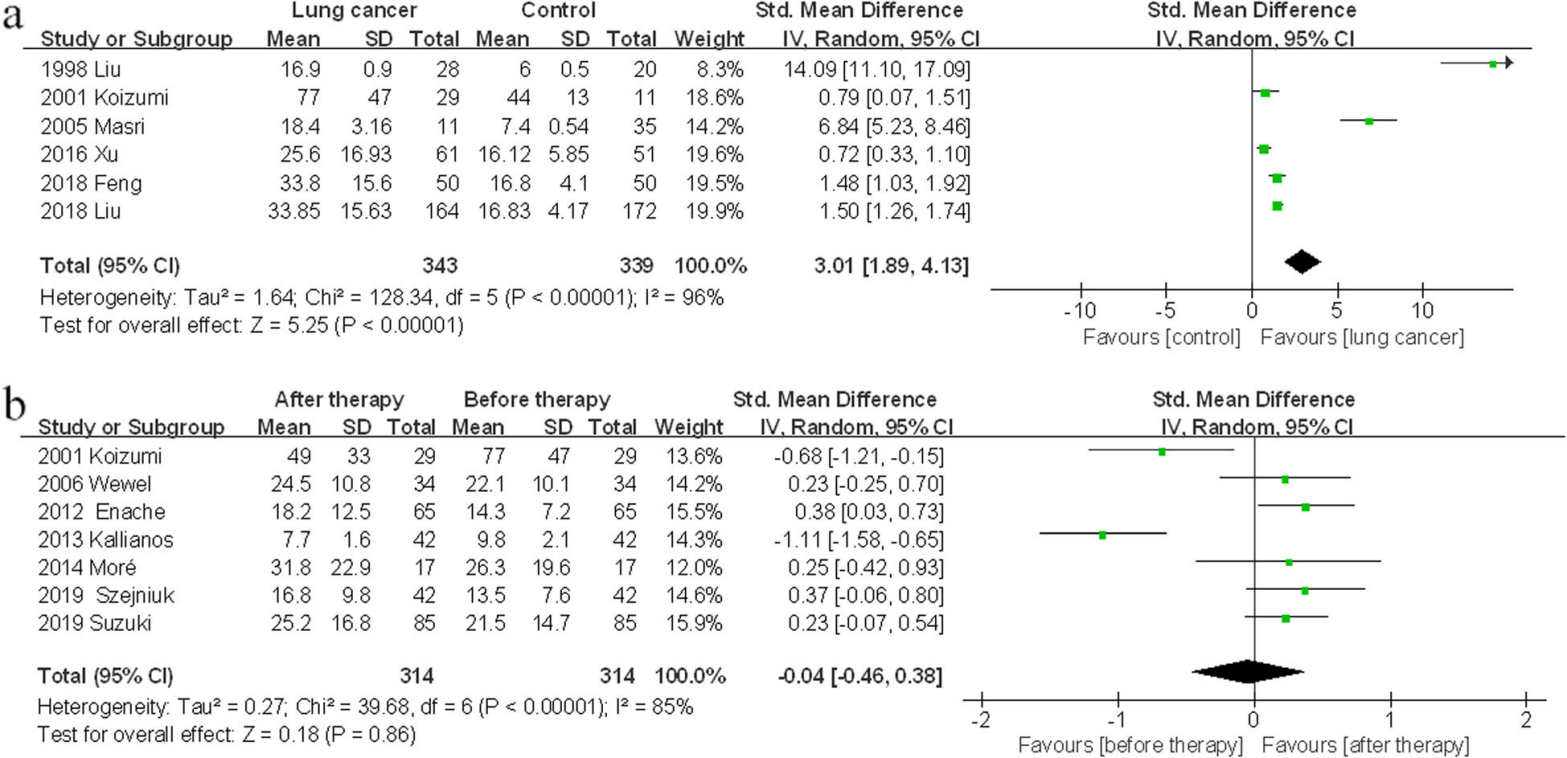
Comparisons of FeNO level between lung cancer patients and healthy subjects, or between pretreatment and posttreatment. **(a)** FeNO level in lung cancer patients and healthy subjects; **(b)** FeNO level in lung cancer patients before and after therapy

There were 6 case-control studies about FeNO level. The FeNO level in lung cancer patients was much higher than that in control subjects (SMD 3.01, 95% CI 1.89–4.13, *p* < 0.00001) (Fig. 2a).

### FeNO level between pretreatment and posttreatment

Seven observational studies reported FeNO level detected before and after therapy. As a result, no significant difference of FeNO level was observed between pretreatment and posttreatment (SMD -0.04, 95% CI -0.46-0.38, *p* > 0.05) (Fig. 2b). However, FeNO level elevated in NSCLC subgroup (SMD 0.28, 95% CI 0.04–0.51, *p* = 0.02) and long-term subgroup (SMD 0.36, 95% CI 0.11–0.61, *p* = 0.005). In other subgroups, there was no evident variance of FeNO level (Table S1).

### Blood NO level between lung cancer patients and control subjects

**Fig. 3:**
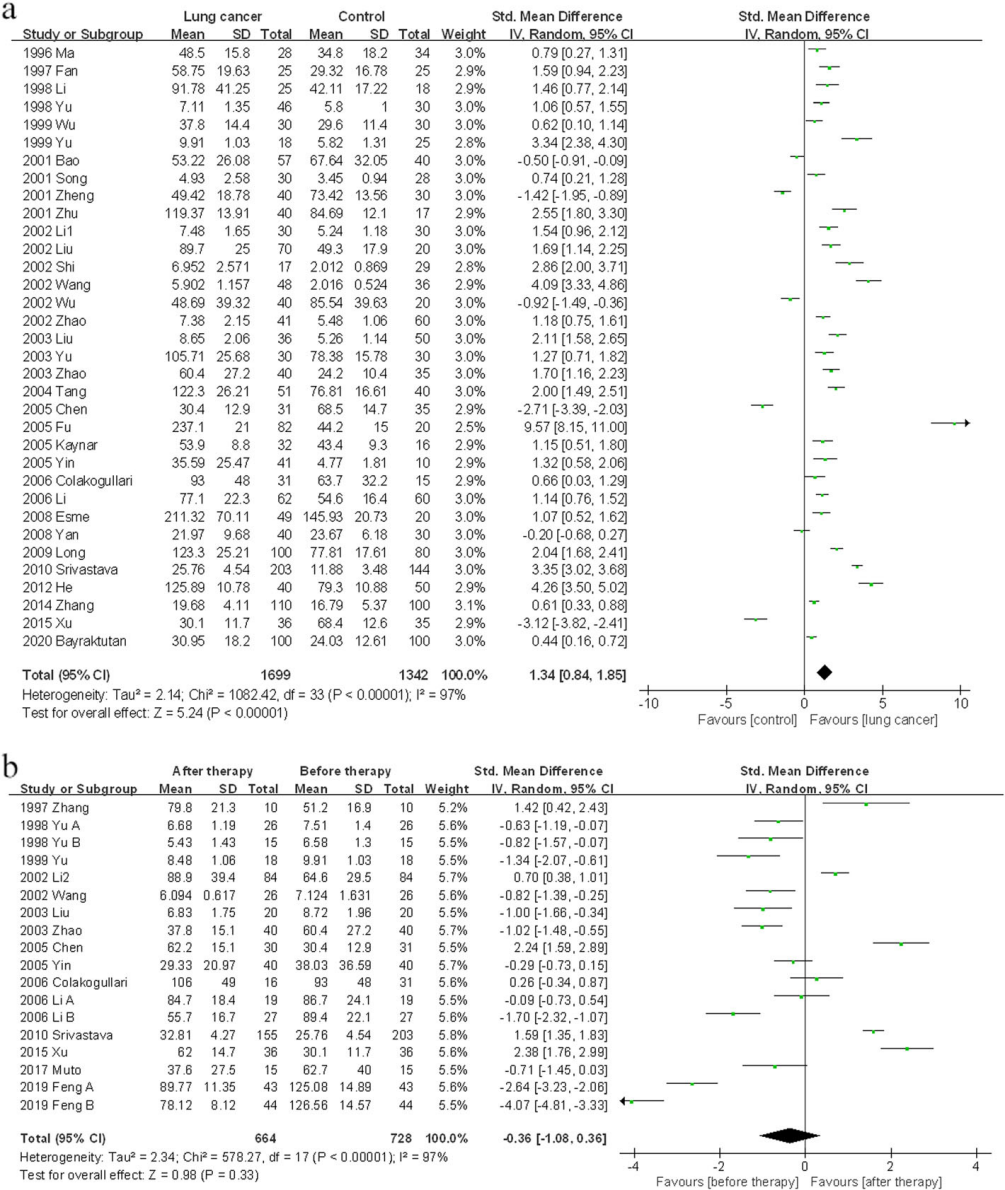
Comparisons of blood NO level between lung cancer patients and healthy subjects, or between pretreatment and posttreatment. **(a)** Blood NO level in lung cancer patients and healthy subjects; **(b)** Blood NO level in lung cancer patients before and after therapy

Thirty-four case-control studies provided data of blood NO level derived from both lung cancer and control groups. Generally, blood NO concentration in lung cancer patients was higher than that in control subjects (SMD 1.34, 95% CI 0.84–1.85, *p* < 0.00001) (Fig. 3a). Subgroup analysis indicated this effect existed in almost all subgroups divided by nationality, sex, age and smoking status. However, this effect was only observed in NSCLC subgroup (SMD 2.40, 95% CI 1.76–3.03, *p* < 0.00001), while in subgroup containing SCLC patients, there was no difference of blood NO level between lung cancer patients and control subjects (SMD 0.38, 95% CI -0.25-1.02, *p* = 0.23). The similar phenomenon was also found in subgroups divided by cancer stage. In the subgroup including both early and advanced stage patients, the blood NO level in patients was higher than that in control subjects (SMD 1.60, 95% CI 0.92–2.27, *p* < 0.00001). For other subgroups (early stage, advanced stage and unknown stage), the difference did not exist (Table S2).

### Blood NO level in lung cancer patients with different pathological types and stages

**Fig. 4:**
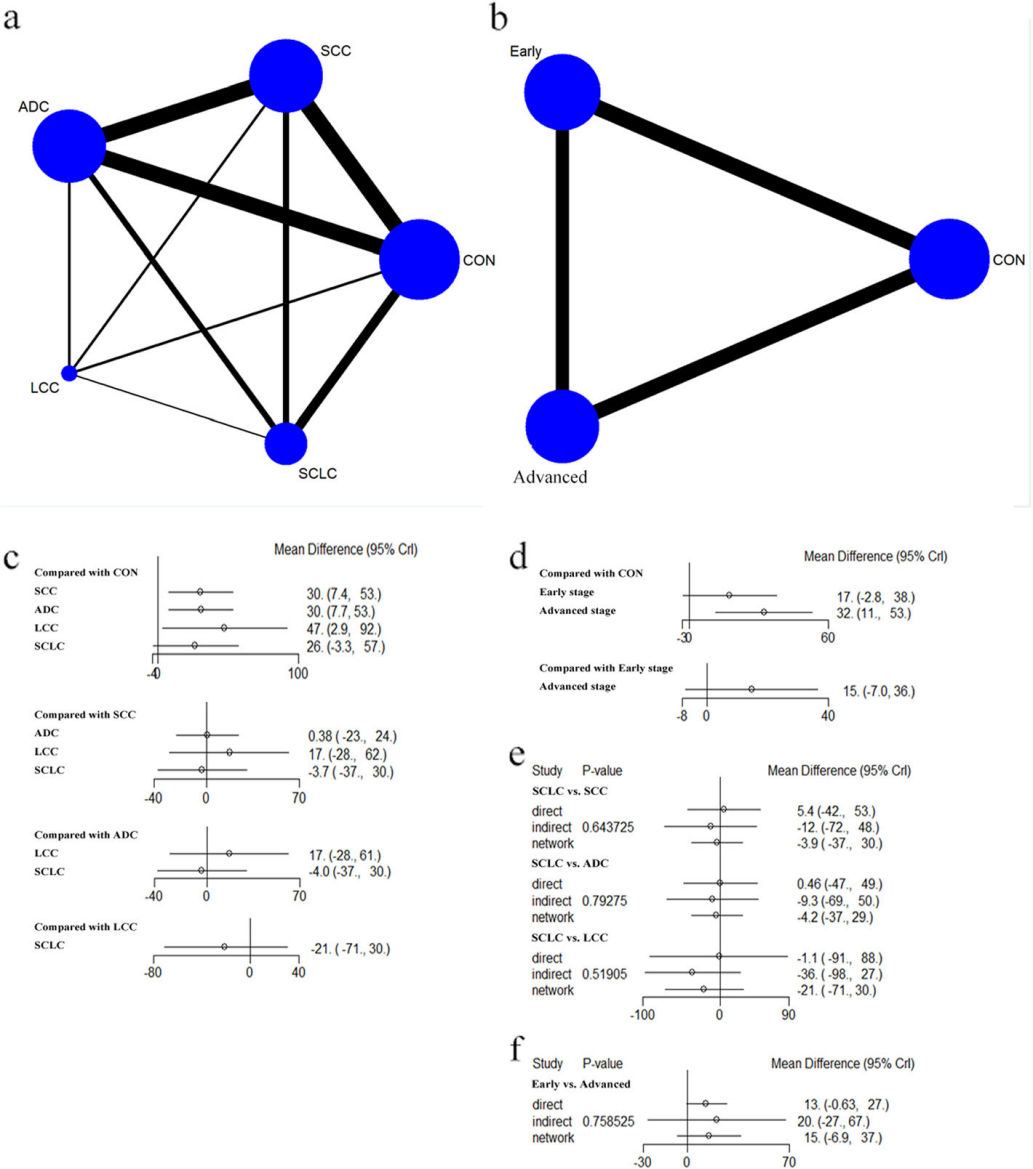
Network meta-analysis of blood NO level in lung cancer patients with different pathological types and stages. **(a)** network plot of lung cancer patients with different pathological types; **(b)** network plot of lung cancer patients with different stages; **(c)** pairwise comparisons of blood NO level in controls and lung cancer patients with different pathological types; **(d)** pairwise comparisons of blood NO level in controls and lung cancer patients with different stages; **(e)** direct and indirect comparisons between two groups with different pathological types (SCC vs. SCLC, ADC vs. SCLC and LCC vs. SCLC); **(f)** direct and indirect comparisons between two groups with different stages (early stage vs. advanced stage)

A total of 16 studies reported the blood NO level of lung cancer patients with different pathological types respectively, including squamous cell carcinoma (SCC), adenocarcinoma (ADC), large cell carcinoma (LCC) and small cell lung cancer (SCLC) (Fig. 4a). To compare the blood NO level in each cancer type, network meta-analysis based on Bayesian random-effect model was applied. As a result, blood NO level in each cancer type except for SCLC was higher than that in control patients. There was no significant difference of blood NO level among four kinds of lung cancer patients (Fig. 4c). The model ranked each pathological type by their relative effect (probabilities of being higher). According to these results, pathological type with the higher probability of being ranked first is LCC, with a probability of 67%, followed by other three kinds of lung cancer, whose probability ranged from 10 to 12%. Cumulative probability of being the pathological type with highest blood NO level was calculated and the cumulative ranking curve of each pathological type was obtained to calculate the SUCRA. According to SUCRA results, LCC (SUCRA = 83.5%) presented as the cancer type with highest blood NO level and followed in order by ADC (58%), SCC (56.5%), SCLC (50%) and control (1.5%) (Table S3). The node-splitting approach allowed for the identification of two inconsistent nodes (SCC vs. SCLC, ADC vs. SCLC and LCC vs. SCLC). No inconsistent results of direct and indirect comparisons were observed in these three pairs of comparisons (Fig. 4e). In addition, Brooks-Gelman-Rubin plot illustrated that the NMA model presented good convergence.

There were 19 studies about the blood NO level of lung cancer patients in different stages (early or advanced) (Fig. 4b). The NMA model revealed that the blood NO level in advanced stage but not early stage was higher than that in control subjects (Fig. 4d). The model ranked each stage by their relative effect, and advanced stage was ranked first, with a probability of 91%. According to SUCRA results, patients in advanced stage (SUCRA = 95.5%) had the highest blood NO level and followed in order by early stage (52.5%) and control (2.5%) (Table S4). The node-splitting approach allowed for the identification of two inconsistent nodes (early stage vs. advanced stage). No inconsistent results of direct and indirect comparisons were observed between these two groups (Fig. 4f). In addition, Brooks-Gelman-Rubin plot illustrated that the NMA model presented good convergence.

### Blood NO level between pretreatment and posttreatment

Fifteen observational studies including 18 cohorts reported blood NO level detected before and after therapy. As a result, no significant difference of blood NO level was observed between pretreatment and posttreatment (SMD -0.36, 95% CI -1.08-0.36, *p* > 0.05) (Fig. 3b). However, blood NO level decreased in NSCLC subgroup (SMD -0.95, 95% CI -1.89-0.00, *p* = 0.05) after therapy. In other subgroups, there was no evident variance of blood NO level (Table S1).

### Publication bias

**Fig. 5:**
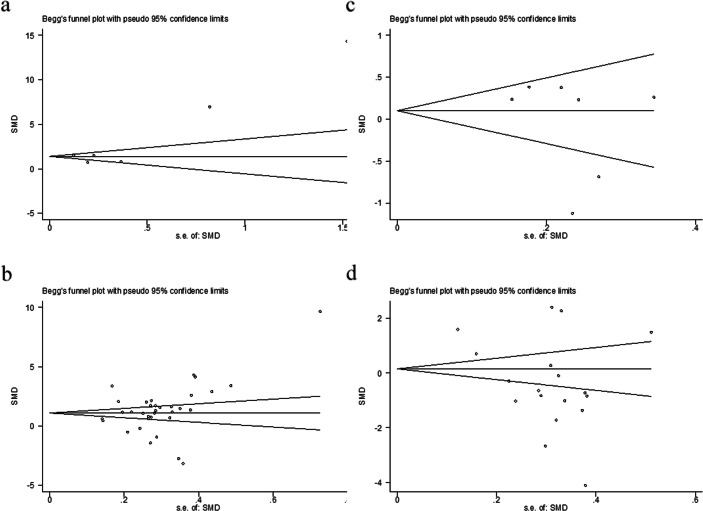
Begg’s funnel plot of comparisons between cases and controls or between pretreatment and posttreatment. **(a)** Begg’s funnel plot of FeNO in case-control studies; **(b)** Begg’s funnel plot of blood NO in case-control studies; **(c)** Begg’s funnel plot of FeNO change in studies comparing FeNO level before and after therapy; **(d)** Begg’s funnel plot of blood NO change in studies comparing blood NO level before and after therapy

Publication bias was tested using Begg’s and Egger’s tests. These tests did not show significant results in comparisons of FeNO. However, Begg’s test and Egger’s test showed significant results in the comparisons of blood NO level between cases and control subjects, and between pretreatment and posttreatment, respectively (Table S5). The distribution of data points revealed asymmetry (Fig. 5). These results indicated the possibility of publication bias.

## Discussion

Lung cancer has becoming a great threat of global health due to its high incidence and mortality [62]. The five-year survival of this disease is relatively low, compared with other kinds of cancers [62]. A large proportion of patients were diagnosed when the lung cancer progressed to advanced stage. Much efforts have been made to diagnose lung cancer as early as possible. Tumor markers are molecules related to various types of tumors, which can be measured in blood as well as other biological samples [5, 63]. The concentrations of these markers are very low in healthy population, but they can elevate dramatically in cancer patients. During the past years, several markers related to lung cancer were widely applied in clinic. However, the sensitivity and specificity were limited in the diagnosis of lung cancer. So, there is an urgent need for novel markers in diagnosing this disease, especially for those at early stage.

NO is a small molecule generated in many kinds of human cells. It was demonstrated that NO was involved in development of lung cancer in basic researches [3, 4, 64, 65]. Emerging studies has been carried out to evaluate whether NO can be regarded as a novel marker for lung cancer. However, controversial results were observed among these researches. In this meta-analysis, we summarized the previous data, and found FeNO value is much higher in lung cancer patients than that in control subjects, which meant an appropriate cut-off point of this index would be attributed to discriminate between lung cancer patients and healthy individuals. In some studies, the authors reported the FeNO values in different pathological types of lung cancer. Never the less, it was not further analyzed that whether the disparity of FeNO level existed among various types of lung cancer, due to the limited number of included literatures. Therefore, it is worthy to compare FeNO level in cancer patients with different pathological types in future studies. In contrast, a large proportion of included studies were about blood NO and lung cancer. As a result, an elevated blood NO level was found in lung cancer patients, which indicated this index was propitious for identifying lung cancer patients from general population. Subgroup analysis suggested ethnicity, sex, age and smoking status did not affect the result. However, the increase of blood NO level was only observed in the subgroup consisting of NSCLC patients, while in another subgroup containing SCLC patients, this phenomenon disappeared. It could be predicted that the rise of blood NO level existed in NSCLC but not SCLC patients, which was further confirmed by network meta-analysis. In addition, NMA results showed no difference in the comparisons of any two kinds of lung cancer types. LCC group seemed to be of highest blood NO value. However, the sample size of LCC group was rather small, subsequently, the results would be further validated in the future. NMA results also revealed that blood NO level in advanced stage but not early stage was higher than that in controls. Moreover, there was no difference of blood NO level between different stages. These results seemed to indicate that this index was less sensitive to capture lung cancer at early stage.

For lung cancer patients, proper treatments help to prolong survival time. Besides traditional treatments such as operation, chemotherapy and radiotherapy, a series of novel treatments have been developed, including molecular target therapy and immunotherapy [66]. In addition, traditional Chinese medicine is also an option of adjuvant therapy in China. As NO is considered to play an important role in lung cancer, the change of NO level between pretreatment and posttreatment has been drawn much attention. In this study, we compared both FeNO and blood NO data collected before and after therapy, and found almost no variance of NO level except for NSCLC and long-term subgroups. FeNO level escalated while blood NO level declined in NSCLC subgroup after treatment. Given that both FeNO and blood NO level increased in lung cancer patients, it seemed to be rather intriguing that the changing trends of FeNO level was just opposite to that of blood NO level after treatment. There were several reasons might lead to such results. First, it should be noted that patients in those two subgroups received different therapy regimens. The changing tendency of NO level was found to be inconsistent under different treatments. For example, Kallianos et al. found FeNO level decreased after chemotherapy [48], while Enache et al. reported FeNO level increased after radiotherapy [47]. So, it was necessary to detect both FeNO and blood NO levels in the same study to prevent the disturbance of therapy regimens. Another point that could not be overlooked was that there was great gap of follow up periods among different studies, from 2 days to more than 2 years [7, 10, 14, 46, 47, 52]. Our results showed that FeNO level increased in long-term follow up subgroup but not in short-term follow up subgroup, while follow up period seemed not affect the pooled results of blood NO. In fact, NO value did not maintain the same level during the whole follow up period. According to the study by Li et al., the blood NO level decreased evidently 3 days after surgery, but increased slightly, and rose to the peak level, which is similar to that detected before therapy, 3 weeks after surgery [39]. In our study, we only chose the NO data collected at the last follow up time point, compared with that collected before therapy. Therefore, it was important to survey kinetic variation of NO level rather than observe NO value at a single time point. Last but not least, NO level would fluctuate under different disease status. As most studies included in our meta-analysis did not report the information of therapy effect or disease control, it could not be excluded that lung cancer progression offset the effect of previous therapy and finally affected the NO level, especially in patients undergone long-term follow up.

Our study had some deficiencies. First, the quantity of lung cancer patients in the included studies was few, especially for LCC patients. The small sample size might affect the stableness of the results. Second, a large proportion of included studies was of low quality, according to NOS. In some studies, the basic characteristics of control subjects (i.e. sex, age) were absent or not matched with lung cancer patients. The unmatched basic conditions might disturb the final results. Third, while most patients enrolled to study were Chinese, the remaining was consisting of other Asian and Caucasian. For Africans, lung cancer is also a common disease. It was regretful that almost no data on Africans was available. The incomprehensive data might affect the reliability of this study. Moreover, smoking is an important risk factor to the development of lung cancer, and it should be well controlled in these studies. However, a part of subjects in several studies have no information of smoking status. It might lead to the confusing results. Last but not least, NO is an indicator of airway inflammation in chronic airway diseases. It has been demonstrated that FeNO is elevated in COPD and asthma patients in previous studies [67–70]. COPD or asthma can be coexisted with lung cancer in some patients. For instance, Suzuki et al. and Wewel et al. reported part of lung cancer patients in their studies were combined with COPD [46, 51]. In contrast, lung cancer patients without comorbidities were recruited in several studies [12, 44, 45]. However, the majority studies did not provide the information that whether lung cancer patients had concomitant diseases. Concomitant disease may be a confounding factor which affected the final results. The deficiency of information about combined disease in lung cancer patients restricted us from further analysis.

## Conclusion

In conclusion, we first demonstrated an evident augment of FeNO and blood NO level existed in whole lung cancer patients, but obvious change of these indexes was confirmed between pretreatment and posttreatment only in NSCLC patients. Our results suggested FeNO and blood NO level would contribute to diagnosis of lung cancer and evaluation of therapy effect, especially for NSCLC patients. Due to the various shortcomings in the present studies, future studies with a larger sample size, covering different pathological types and stages, and proper selection of control subjects should be carried out to further validate the relationship between NO and this disease.

## Supplementary Information

**Additional file 1.**

## Data Availability

The datasets used in this study were available from the corresponding author upon reasonable request.

## Abbreviations

NO: Nitric oxide
FeNO: Fraction of exhaled nitric oxide
SMD: Standard mean difference
CI: Confidence interval
NMA: Network meta-analysis
SUCRA: Surface under the cumulative ranking curve
NSCLC: Non- small cell lung cancer
SCC: Squamous cell carcinoma
ADC: Adenocarcinoma
LCC: Large cell carcinoma
SCLC: Small cell lung cancer
