# Supplementary material for: Nitric oxide in occurrence, progress and therapy of lung Cancer: a systemic review and meta-analysis
Source: BMC Cancer. 2021 Jun 8;21:678. doi: 10.1186/s12885-021-08430-2 (PMC8188673; doi:10.1186/s12885-021-08430-2)
Supplement: Supplementary file 1 — Additional file 1. [file 12885_2021_8430_MOESM1_ESM.docx]

Nitric Oxide in Occurrence, Progress and Therapy of Lung Cancer: A Systemic Review and Meta-analysis

Hongbin Zhou^1, #^, Jiuke Li^2, #^, Zhewen Chen^3^, Ying Chen^3^, Sa Ye^1, 3, *^

1 Department of Respiratory Medicine, Zhejiang Provincial People’s Hospital, Affiliated People’s Hospital, Hangzhou Medical College, Hangzhou, Zhejiang, China

2 Department of Ophthalmology, Hangzhou Aier Eye Hospital, Hangzhou, Zhejiang, China

3 Department of Nutrition, Zhejiang Provincial People’s Hospital, Affiliated People’s Hospital, Hangzhou Medical College, Hangzhou, Zhejiang, China

# Two authors (Hongbin Zhou and Jiuke Li) contributed equally to this work.

* Correspondence author: Sa Ye, Department of Respiratory Medicine, Department of Nutrition, Zhejiang Provincial People’s Hospital, Affiliated People’s Hospital, Hangzhou Medical College, 158 Shangtang Road, Hangzhou, 310014, China; Email: yesa@hmc.edu.cn

TableS1 Subgroup analysis of pooled results for FeNO and blood NO variation after therapy in enrolled studies

| **Subgroup** | FeNO | | | |  | Blood NO | | | |
| --- | --- | --- | --- | --- | --- | --- | --- | --- | --- |
|  | No. of studies | SMD [95% CI] | P | I^2^ |  | No. of studies | SMD [95% CI] | P | I^2^ |
| **Country** | | | | | | | | | |
| Eastern countries | 2 | -0.20 [-1.09, 0.70] | 0.67 | 88% |  | - | - | - | - |
| Western countries | 5 | 0.02 [-0.55, 0.60] | 0.94 | 87% |  | - | - | - | - |
| China | - | - | - | - |  | 15 | -0.51 [-1.29, 0.26] | 0.20 | 96% |
| Non-China | - | - | - | - |  | 3 | 0.41 [-0.99, 1.82] | 0.56 | 96% |
| **Pathology** | | | | | | | | | |
| NSCLC | 3 | **0.28 [0.04, 0.51]** | **0.02** | 0 |  | 12 | **-0.95 [-1.89, 0.00]** | **0.05** | 98% |
| NSCLC+SCLC | 4 | -0.29 [-1.02, 0.44] | 0.44 | 91% |  | 4 | 0.92 [-0.37, 2.21] | 0.15 | 94% |
| Unknown | - | - | - | - |  | 2 | 0.60 [-2.59, 3.79] | 0.71 | 98% |
| **Stage** | | | | | | | | | |
| Early + advanced | 4 | 0.10 [-0.34, 0.54] | 0.65 | 75% |  | 4 | -0.40 [-1.29, 0.49] | 0.38 | 93% |
| Early | - | - | - | - |  | 4 | 0.11 [-1.25, 1.47] | 0.87 | 96% |
| Advanced | 1 | 0.23 [-0.07, 0.54] | 0.13 | NA |  | 8 | -0.83 [-2.37, 0.70] | 0.29 | 98% |
| Unknown | 2 | -0.45 [-1.79, 0.88] | 0.51 | 91% |  | 2 | 0.69 [-2.61, 4.00] | 0.68 | 98% |
| **Treatment** | | | | | | | | | |
| Radiotherapy | 4 | 0.10 [-0.38, 0.58] | 0.69 | 75% |  | - | - | - | - |
| Chemotherapy | 2 | -0.45 [-1.76, 0.87] | 0.51 | 94% |  | 11 | -0.10 [-0.98, 0.77] | 0.82 | 97% |
| Immunotherapy | 1 | 0.23 [-0.07, 0.54] | 0.13 | NA |  | - | - | - | - |
| Surgery | - | - | - | - |  | 6 | -0.23 [-1.16, 0.70] | 0.63 | 94% |
| Comprehensive therapy | - | - | - | - |  | 1 | -4.07 [-4.81, -3.33] | <0.0001 | NA |
| **Follow up** | | | | | | | | | |
| Long term | 3 | **0.36 [0.11, 0.61]** | **0.005** | 0 |  | 2 | 0.47 [-1.79, 2.72] | 0.68 | 97% |
| Short term | 1 | 0.23 [-0.25, 0.70] | 0.35 | NA |  | 10 | -0.70 [-1.70, 0.31] | 0.17 | 96% |
| Unknown | 3 | -0.51 [-1.39, 0.38] | 0.26 | 92% |  | 6 | -0.08 [-1.09, 0.94] | 0.88 | 95% |

TableS2 Subgroup analysis of pooled results for blood NO in case-control studies

| Subgroup | No. of studies | SMD [95% CI] | P | I^2^ |
| --- | --- | --- | --- | --- |
| **Country** | | | | |
| China | 29 | **1.35 [0.79, 1.90]** | **<0.00001** | 97% |
| Non-China | 5 | **1.34 [0.01, 2.67]** | **0.05** | 98% |
| **Age** | | | | |
| Age matched | 16 | **1.39 [0.78, 1.99]** | **<0.00001** | 96% |
| Age unmatched | 6 | **1.77 [0.56, 2.97]** | **0.004** | 97% |
| Unknown | 12 | 1.11 [-0.05, 2.27] | 0.06 | 98% |
| **Sex** | | | | |
| Sex matched | 23 | **1.78 [1.25, 2.30]** | **<0.00001** | 96% |
| Sex unmatched | 7 | **1.47 [0.38, 2.57]** | **0.008** | 97% |
| Unknown | 4 | -1.52 [-3.18, 0.15] | 0.07 | 96% |
| **Smoking** | | | | |
| Smoking matched | 3 | **0.92 [0.49, 1.36]** | **<0.0001** | 64% |
| Smoking unmatched | 1 | **0.44 [0.16, 0.72]** | **0.002** | NA |
| Unknown | 30 | **1.42 [0.82, 2.02]** | **<0.00001** | 97% |
| **Pathology** | | | | |
| NSCLC | 16 | **2.40 [1.76, 3.03]** | **<0.00001** | 96% |
| NSCLC+SCLC | 14 | 0.38 [-0.25, 1.02] | 0.23 | 95% |
| Unknown | 4 | 0.40 [-1.68, 2.48] | 0.70 | 98% |
| **Stage** | | | | |
| Early + advanced | 19 | **1.60 [0.92, 2.27]** | **<0.00001** | 96% |
| Early | 3 | 0.77 [-2.61, 4.14] | 0.66 | 99% |
| Advanced | 2 | 2.02 [-0.62, 4.66] | 0.13 | 98% |
| Unknown | 10 | **0.91 [0.15, 1.66]** | **0.02** | 97% |

TableS3 Summary of ranks and SUCRA in controls and lung cancer patients with different pathological types

| Rank | CON | SCC | ADC | LCC | SCLC |
| --- | --- | --- | --- | --- | --- |
| 1 | 0 | 0.1 | 0.11 | 0.67 | 0.12 |
| 2 | 0 | 0.31 | 0.33 | 0.13 | 0.23 |
| 3 | 0 | 0.35 | 0.34 | 0.09 | 0.21 |
| 4 | 0.06 | 0.23 | 0.21 | 0.09 | 0.41 |
| 5 | 0.94 | 0 | 0 | 0.02 | 0.04 |
| **SUCRA** | **0.015** | **0.565** | **0.58** | **0.835** | **0.5** |
| **SUCRA Rank** | **5** | **3** | **2** | **1** | **4** |

Abbreviations: CON – Control; SCC – Squamous cell carcinoma; ADC – Adenocarcinoma; LCC – Large cell carcinoma; SCLC – Small cell lung cancer

TableS4 Summary of ranks and SUCRA in controls and lung cancer patients with different stages

|  | Control | Early stage | Advanced stage |
| --- | --- | --- | --- |
| 1 | 0 | 0.09 | 0.91 |
| 2 | 0.05 | 0.87 | 0.09 |
| 3 | 0.95 | 0.05 | 0 |
| **SUCRA** | **0.025** | **0.525** | **0.955** |
| **SUCRA Rank** | **3** | **2** | **1** |

TableS5 Publication bias in meta-analysis: comparisons between cases and controls or between pretreatment and posttreatment

| Comparisons | FeNO | |  | Blood NO | |
| --- | --- | --- | --- | --- | --- |
|  | Begg‘s test | Egger's test |  | Begg‘s test | Egger's test |
| Controls vs. Cases | 0.26 | 0.111 |  | 0.047 | 0.298 |
| Pretreatment vs. Posttreatment | 0.23 | 0.401 |  | 0.363 | 0.019 |
